# Justice-centered best practices for accessibility to public buildings in a tier II city: Insights from a Delphi expert consensus

**DOI:** 10.12688/f1000research.156920.1

**Published:** 2024-10-07

**Authors:** Sidhiprada Mohapatra, G Arun Maiya, Ullas U Nayak, Rashmi Sheelvant, Vennila J, Joanne Watson, Rama Devi Nandineni

**Affiliations:** 1 Centre for Comprehensive Rehabilitation, Department of Physiotherapy, Manipal College of Health Professions, Manipal Academy of Higher Education, Manipal, Karnataka, 576104, India; 2 Centre for Podiatry & Diabetic Foot Care and Research, Department of Physiotherapy, Manipal College of Health Professions, Manipal Academy of Higher Education, Manipal, Karnataka, 576104, India; 3 Division of Anatomy, Department of Basic Medical Sciences, Manipal Academy of Higher Education, Manipal, Karnataka, 576104, India; 4 Department of Physiotherapy, Manipal College of Health Professions, Manipal Academy of Higher Education, Manipal, Karnataka, India; 5 Statistics, Manipal College of Health Professions, Manipal Academy of Higher Education, Manipal, Karnataka, 576104, India; 6 School of Health and Social Development, Institute for Health Transformation, Deakin University, Burwood, Victoria, 3125, Australia; 7 Manipal School of Architecture and Planning, Manipal Academy of Higher Education, Manipal, Karnataka, 576104, India

**Keywords:** administration, decision tree, expert, inclusion, municipality, machine learning, public spaces

## Abstract

**Background:**

Despite beneficial progress in policies, awareness and advocacy, accessibility gaps exist in public buildings in India. Challenges achieving full inclusivity still exist, due to a lack of clear guidance for implementing accessible solutions. Retrofitting older buildings, particularly in developing tier II cities is a major challenge. The authors of this paper aimed to address this issue using a four-round Delphi method to generate a Justice-Centered Best Practices (JCBPs) for accessibility provisions for individuals with mobility disabilities.

**Methods:**

Conducted in Udupi, the study involved experts including administrators, policy implementers, auditors, advocates, healthcare professionals, individuals with disabilities and their caregivers. In the first round, a 117-item list was generated through triangulation of three methods. In subsequent rounds, experts rated each item using a 5-point Likert scale on feasibility, affordability and priority. Responses were considered valid if the agreement reached ≥80% on the total score. The prioritised list of JCBPs was finalised at a consensus meeting.

**Results:**

Out of forty-eight experts who began the study, 16 participated in the final meeting. The Wilcoxon signed rank test (p value>0.05) of expert ranking indicated that the scoring of items remained consistent between the two rounds. A machine learning decision tree analysis identified items securing ≥ 80% agreement as the most reliable decision with an accuracy=71.43%. The McNemar’s Test p value=0.79 confirmed consistency of expert scoring on the items with high agreement rates.

**Conclusion:**

Finally, 33 built and non-built environment items scored highest rank. Stakeholder engagement, use of low-cost technology solutions, coordination between public administrations, funding, good governance practice, awareness, and advocacy were few of the solutions that can help ensure accessibility is in place for individuals with mobility disabilities. The study methodology and findings create a robust foundation for evidence-based JCBPs for accessibility provisions for individuals with mobility disabilities.

## Introduction

Globally, the number of individuals living with disabilities is rising. The causes and consequences of disabilities vary across different stages of life and among different nations. ^1, 2^ For instance, in the United States, over 24.6% of individuals with disabilities are in the labor force, ^3^ while in India, individuals with disabilities continue to be marginalised and underrepresented in the workforce. ^4^ The lack of accessible infrastructure and unequal opportunities for education and skill development prevent individuals with disabilities from participating equitably in paid employment. ^2^

International protocols such as the Standard Rules on the Equalization of Opportunities for Persons with Disabilities, the United Nations, and the Universal Declaration of Human Rights (UDHR) acknowledge the necessity of promoting and protecting the rights of individuals with disabilities, with the duty resting on governments and governing bodies. ^5^ Globally, efforts are being undertaken towards the development of inclusive nations. The Sustainable Development Goals (SDG), encourages all states to eliminate poverty and enhance living conditions by providing equal opportunities, and equitable environment for all. SDG 11, in particular, promotes inclusive, safe, sustainable, and resilient cities. ^6^ With 60% of the world’s population residing in cities, fostering inclusive cities are likely to have a positive impact on both health and quality of life. ^7^ This emphasizes the need to recognize the opportunity divide and economic costs associated with the exclusion of individuals with disabilities from mainstream society. ^6, 7^

Despite inclusive practices gaining global momentum, most developing nations, including India, have yet to establish such practices as mainstream. Consequently, individuals with disabilities remain a minority group in education and the workforce, leading to dependency, poverty and exclusion, further limiting access to services. ^4, 8, 9^ In India, 40-42% of individuals with disabilities face poverty, affecting their social participation in leisure, political, cultural and religious spheres. ^4, 10, 11^ This creates a cascading effect of disability, marginalisation and lack of social participation. ^12^ Accessible public buildings are crucial for promotion of equal opportunities and prevention of discrimination against individuals with disabilities. ^10^ Public buildings serve as providers of services to all, and aid in mitigating the symbiotic relationship between poverty, exclusion and disability. ^4, 10^ Research from developed and developing countries have identified challenges experienced by individuals with disabilities due to inaccessible public buildings and facilities. ^13^ However, there is a significant gap in knowledge transfer and implementation in long-neglected tier II cities. ^14^ These cities are characterized by diverse settlements, governance systems, multiple infrastructural developments, differing cultural and social constructs, and varied economic opportunities. ^14– 16^ These features necessitate a multidisciplinary approach to address their unique accessibility challenges.

Building developers and industries, are often reluctant to implement accessibility provisions, due to perceived costs, lack of user demand, insufficient understanding of user needs and aesthetic concerns. ^17– 20^ They frequently prioritize immediate profits over the long-term value of the building. ^17, 21^ Therefore, it is imperative to utilize collaborative research techniques like Delphi methods that can effectively involve a multidisciplinary team with diverse views, including architects, urban planners, builders, evaluators, and accreditors of civic structures. ^22, 23^

Thus, to address the intricate challenges and engage meaningfully with the end-users we employed the Delphi method. This systematic and structured research method is used in complex situations to elicit anonymous responses from experts. Involving multidisciplinary stakeholders, will aid in building consensus and proposing strategic solutions for accessibility challenges. ^24^ Local authorities can utilize these insights to create inclusive cities by understanding the needs of all stakeholders and allocating resources effectively.

**Aim:** Thus, we used a Delphi consensus process to develop a list of justice-centered best practices to enhance accessibility to public buildings by persons with motor disabilities in Udupi city.

### Theoretical underpinnings

**Figure 1.**
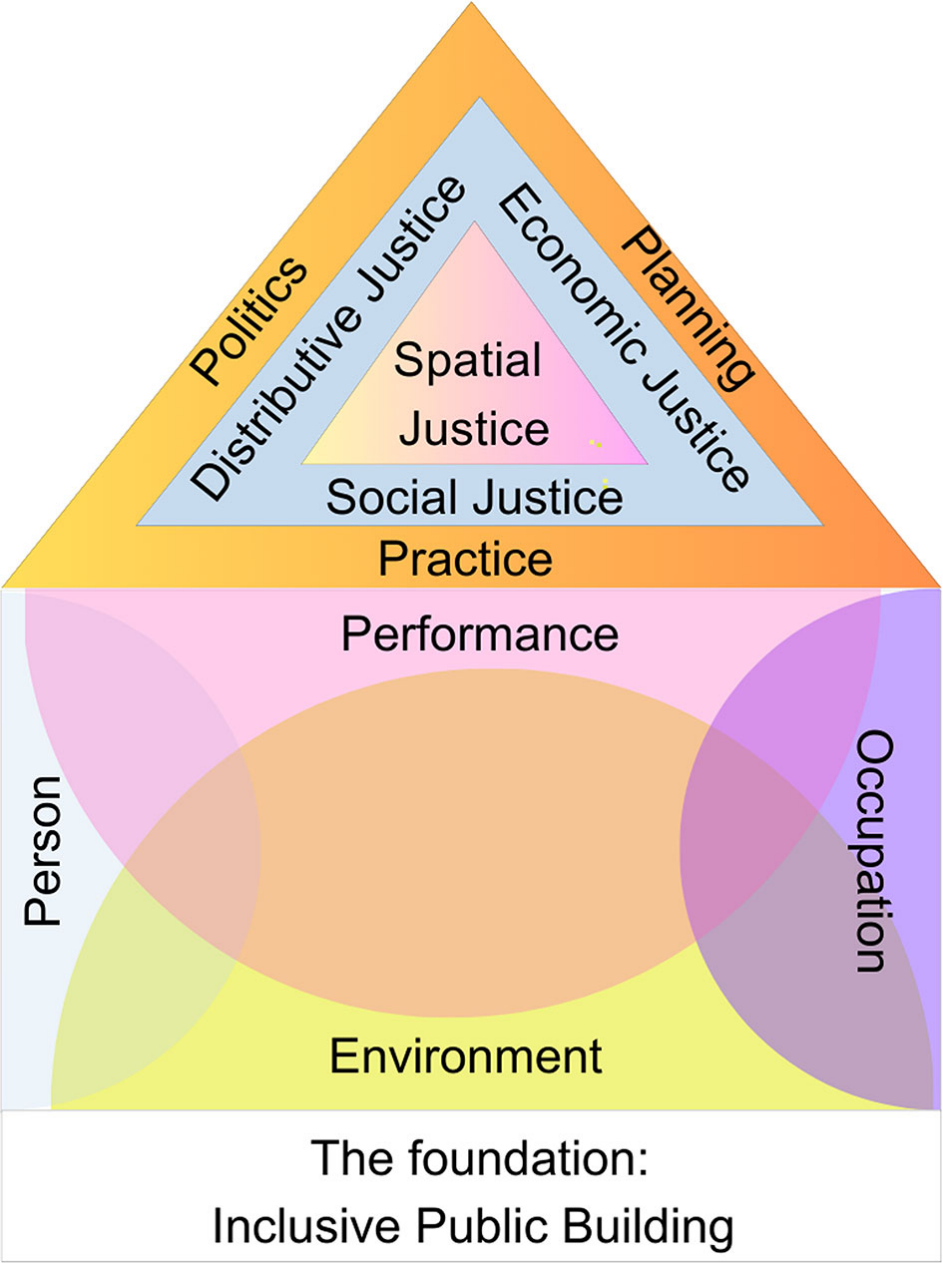
Theoretical framework for the research.

This study is underpinned by two theoretical paradigms, Spatial Justice and the Person-Environment-Occupation-Performance (PEOP) paradigms. An integration of the two frameworks allowed an examination of accessibility to public buildings from a justice-centered perspective. A spatial justice paradigm promotes equitable distribution of resources, advocating for inclusive design and community involvement. ^25^ While, the PEOP framework, enables an examination of the dynamic interaction between individuals with disabilities, their environments, and their activities, focusing on enhancing performance and participation. ^26^ Combining these frameworks provides multidisciplinary practitioners an understanding of spatial inequalities and injustices enabling them to design and manage public buildings that promote inclusivity, accessibility, fairness and enhance performance. Figure 1 illustrates the theoretical frameworks of the research process. This approach provides a framework for considering public buildings in terms of their accommodation of diverse needs, promotion of full participation and equitable access for individuals with mobility disabilities.

## Methods

This study is conducted and reported as per the Recommendations for the Conducting and REporting of DElphi Studies (CREDES) ^27^ as given under reporting guidelines. ^28^

### Case study site

Udupi, a tier II city situated in the southwestern state of Karnataka, India, is renowned for its beaches, temples, and cuisine. The city is known for its extensive history and cultural heritage, which is evident in its architectural style, artwork, and festivities. Furthermore, Udupi is a center of excellence for education and healthcare. The town houses several hospitals with state-of-the-art medical facilities, making it a popular choice for medical tourism. Udupi’s unique blend of tradition and modernity, coupled with its exceptional cultural and natural beauty, make it one of India’s most visited destinations.

**Figure 2.**
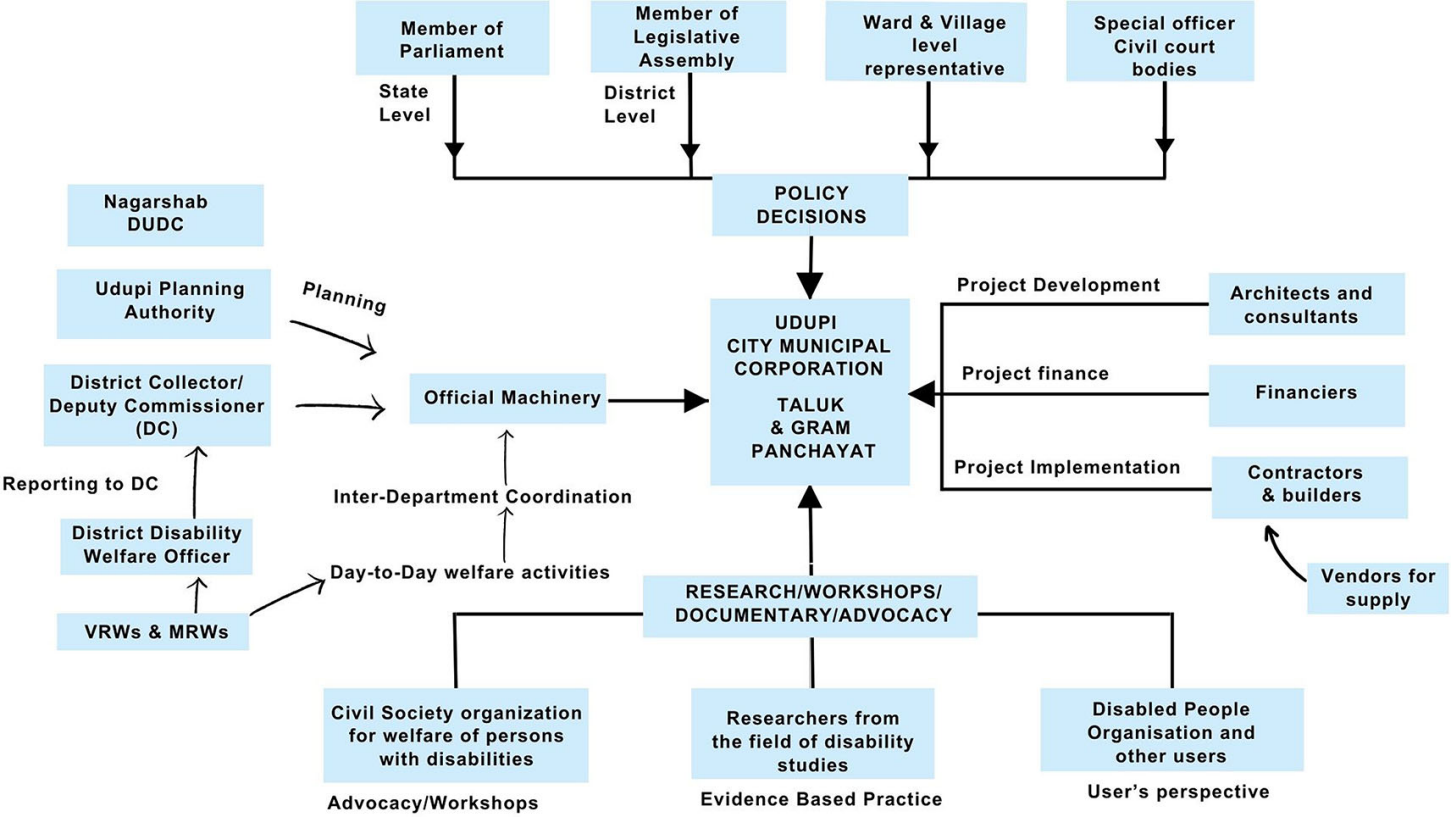
Organizational structure of stakeholders involved in accessibility provisions for public buildings in Udupi city.

The Census of 2011 has classified the urban settlement of Udupi as a Statutory Town. ^29^ The city is governed by the Udupi City Municipal Council, which is entrusted with the provision of fundamental civic amenities to the inhabitants. ^30^ These include water supply, sanitation, waste management, and road maintenance. The Udupi City Municipal Council is further organized into several administrative departments, including the engineering department, which oversees infrastructure development. Organizational structure of stakeholders involved in accessibility provisions for public buildings is shown in Figure 2.

### Research design

A four-round Delphi method was used in the study. The Delphi process is a useful methodology for proposing solutions for future scenarios. It facilitates the contribution of experts’ opinions anonymously, leading to consensus on a phenomenon. The Delphi methodology was chosen for its ability to achieve consensus in domains characterized by uncertainty. ^24^

### Sampling of experts

A sampling frame was generated to list out all experts as per the organogram given in Figure 2. Participants were identified using convenience sampling. Stakeholders involved in city development were invited to participate in the study. This included those managing public facilities and buildings, overseeing public projects, making policy decisions, planning and executing accessibility policies, working in public administration in the city Municipal Council and advocating for disability rights. Further, using a snowball sampling technique, individuals were prompted to suggest other pertinent stakeholders who were involved in matters of accessibility or disability. Additionally, the network that was established through the Centre for Comprehensive Rehabilitation at the academic institution was employed to reach out to all potential stakeholders. Upon confirmation, the purpose of the study, time, and the process of the Delphi technique was explained. Forty-eight experts expressed a willingness to participate. Extended file 1 gives the network of experts. ^31^

### Inclusion and exclusion criteria

To ensure diverse involvement, the researchers approached experts who are engaged in financing, policy-making, administration, standardization, and legal compliance of public buildings, as well as individuals with mobility impairments who utilize these structures. To be considered as an expert, they should understand the needs of individuals with mobility disabilities, and/or should have experience in advocating for disability rights. Table 1 shows the criteria for selection of experts.

**Table 1.** Inclusion and exclusion criteria for selection of experts and users.

Inclusion criteria
1. Individuals who identify themselves as having mobility disability and are community ambulators with or without assistive devices
2. Engaged in governmental or non-governmental disability rights for individuals with mobility disabilities
3. Engaged in governmental administration, policy making, policy implementation, and such activities concerning public buildings
4. People with experiences as constructors, engineers, contractors, builders, architects or other technical disciplines working in private and public sectors.
Exclusion criteria
1. Unwilling to participate in Delphi due to time constraints, or confidentiality challenges

### Data collection

The four rounds of Delphi were conducted in person. All the instructions were given in both English and Kannada. The study was carried out between November 2021 to November 2023. The Delphi rounds are described below.

Round-1: Delphi Questionnaire Development

The first round of the Delphi study included three methods to generate an exhaustive list of needs for individuals with mobility disabilities while accessing public buildings. The list also consists of practices to improve accessibility to public buildings.:

Literature review

The researchers undertook a literature search in PubMed, Scopus, Web of Science, and Cochrane using the terms “mobility disability”, “accessibility”, “experiences”, “built environment” and “public buildings”. We utilized the PEOP model to explore the factors that influence accessibility and task performance in public buildings by persons with motor disabilities. Two authors (SM and UN) reviewed challenges and opportunities to enhance accessibility to public buildings from the literature. All practices including the built environment and non-built environment were included. Extended file 2 gives the search strategies used for the databases. ^32^

Onsite observation

Six public buildings were selected through purposive sampling ensuring a heterogeneous user, substantial pedestrian traffic, and security concerns are met. Unobtrusive observations helped to identify real-time challenges faced by individuals with mobility disabilities, offering insights into problems in a realistic setting. ^33^

Expert inputs

Following this, the experts were requested to enumerate all requisite amenities and arrangements imperative to render public buildings in the city of Udupi entirely accessible to individuals with mobility disabilities. The instructions given were, “Kindly list all requisite amenities and arrangements imperative to render public buildings in the city of Udupi entirely accessible to individuals with mobility disabilities.” Content analysis of the responses was done to develop items for the subsequent rounds using the software Open Coding System 4.0 (ITS and Epidemiology, University of Umea). ^34^ The responses were listed and analysed to identify duplicate responses. Items with similar meanings were also grouped together.

Triangulation and data compilation

To ensure credibility, reliability and trustworthy scientific accuracy, triangulation of the three data sources was adopted. ^35^ To develop the Delphi questionnaire, data captured from all three sources, were systematically compiled. An Excel (Microsoft Inc) spreadsheet was used to collate the data from the literature search, onsite observations, and expert-enumerated items into a comprehensive list. Using the Open Coding System 4.0 ^34^ the compiled data were systematically coded and categorised. In this process, SM, UN and RS mapped all the items, removed duplicates, grouped similar items, and identified themes as accessibility indicators. The refined list of items was then formatted into a structured Delphi questionnaire, ensuring clarity and relevance for the experts. The instructions and items were provided in English to accommodate all experts in Round 2.

Round-2: Delphi questionnaire rating

In this round, experts were asked to rank the items identified from Round-1 using a 5-point Likert scale based on three criteria: affordability, feasibility, and priority. Each item was scored on a scale of 1 to 5, where 1 represented “least score” and 5 represented “highest score.” The total score for each item was generated by summing the scores across all three criteria. The instructions provided were: “Each of the items listed below is imperative to render public buildings in the city of Udupi entirely accessible to individuals with motor disabilities. We kindly request that you score each aforementioned element according to its relative significance, utilizing a 5-point Likert scale.” Extended file 3 provides the original scoring sheet given to experts. ^36^

After collecting the ratings from experts, we analysed the items using descriptive statistics to calculate the mean, median scores, and agreement percentages for each item. Despite the possibility of ranking an item as “1- least score,” none of the items were excluded or rearranged in this round. Also, items with low consensus were also retained until the final meeting to ensure discussion and evaluation of all items.

Round-3: Delphi questionnaire rating

In this round, the same group of 35 experts were contacted with the same set of 117 items. We provided the overall ranking data from Round-2 with the possibility of ranking items differently in this round. The experts were asked to score all the items using the same Likert scale as in Round-2. The experts were prompted to give reasons for any disagreement, and it was recorded. Despite potential changes in rankings, all items were retained for the final consensus meeting to ensure comprehensive evaluation.

### Statistical analysis

For Round 2 and 3, Microsoft Excel 2016 was used to assess the descriptive statistics including mean, median, interquartile range (IQR), ranking and agreement. IQR was to determine the level of agreement on the 5-point scale for each item on the questionnaire.

To compare the scores between these rounds, we performed the Wilcoxon signed-rank test to determine if there were statistically significant differences between the two rounds thereby check stability in ratings by experts. The p values from this test indicated whether the changes in scores were significant. Additionally, we calculated the Kappa statistic to measure the level of agreement between the two rounds. This was done using Jamovi Project 1.4. ^37^ The order of items presented to experts remained fixed.

To identify key factors influencing the rankings and provided a visual representation of the decision-making process among the experts, we utilized R programming language with the ‘rpart’ package to develop a Decision Tree. ^38^ Although the use of machine learning was not initially included in the study protocol, it was later incorporated to strengthen the validation of the expert scoring process. This helped identifying and validating items with the highest agreement, ensuring a robust and reliable final set of recommendations. To develop the Decision Tree the following steps were used:

Data Preparation: The dataset included the total scores for each item on Affordability, Feasibility, and Priority from both Round-2 and 3. Each item was categorized as either “acceptable” or “not acceptable” based on a threshold score (i.e. items with a score above 12 out of 15 were considered acceptable).

Model Building: We first assessed all 117 items using the Decision Tree to identify the main factors influencing the rankings in both rounds. We then focused on items with high agreement (>80%) to build a more targeted Decision Tree, highlighting the most consistently prioritized items.

Validation and Pruning: Cross-validation were performed to validate the model and assess its performance

Output and Interpretation: The Decision Tree output included nodes that represent decision points and branches that represent the outcomes based on specific criteria. Each terminal node (leaf) of the tree provided the final categorization of items as acceptable or not acceptable based on the experts’ ratings.

Statistical measure used for evaluating the performance of Decision Tree analysis are Accuracy (Acc), 95% Confidence Interval (CI), No Information Rate (NIR), P-Value [Acc > NIR] Kappa, McNemar’s Test p value, Sensitivity, Specificity, Positive Predictive Value (PPV), Negative Predictive Value (NPV), Detection Rate, Detection Prevalence, and Balanced Accuracy. Extended file 4 gives the Decision tree coding. ^39^

Round-4: Consensus meeting

All experts from Round-3 were invited to the consensus meeting, which was conducted as two focus group discussions (FGDs). Sixteen experts consented to participate in the final discussion. The 117 items generated from the prior rounds were independently sorted by three researchers (SM, RS, and UN) according to their average rank from both rounds and presented to the experts. The top 40 items, with an agreement of over 80%, were presented separately. If any constituent items were contradictory or included multiple strategies, a common neutral statement was created to facilitate discussion among the experts.

As a result of the discussions, the number of items was reduced to a final list, with some items coalesced by the consensus group and others removed for not reaching the required consensus level. The final consensus statements were crafted to reflect the experts’ agreement on the most critical practices for enhancing accessibility to public buildings for individuals with mobility disabilities

#### Data analysis

Data from FGDs was used to refine and group the items. The final consensus statements were generated, reflecting the experts’ agreement using thematic analysis. These statements were designed to encapsulate all relevant items and address any contradictions or multiple strategies identified during the discussions.

By combining qualitative insights from the FGDs with the quantitative agreement data from previous rounds, the final set of best practices was developed. These practices provide a comprehensive guide for enhancing accessibility to public buildings for individuals with mobility disabilities in Udupi.

### Ethical considerations

The study was approved by the Institutional Research Committee, Manipal College of Health Professions, and the Institutional Ethical Committee-Kasturba Hospital, Manipal (IEC 769/2017) to perform the study. The date of approval from ethics committee was received on 14 ^th^ November 2017. The study period was approved from 14th November 2017 to 30th November 2024. The study was registered under the Clinical Trial Registry- India (CTRI/2018/07/014763). The study adheres to the Declaration of Helsinki. A written informed consent was obtained from experts detailing nature of study and their involvement.

## Results

**Figure 3.**
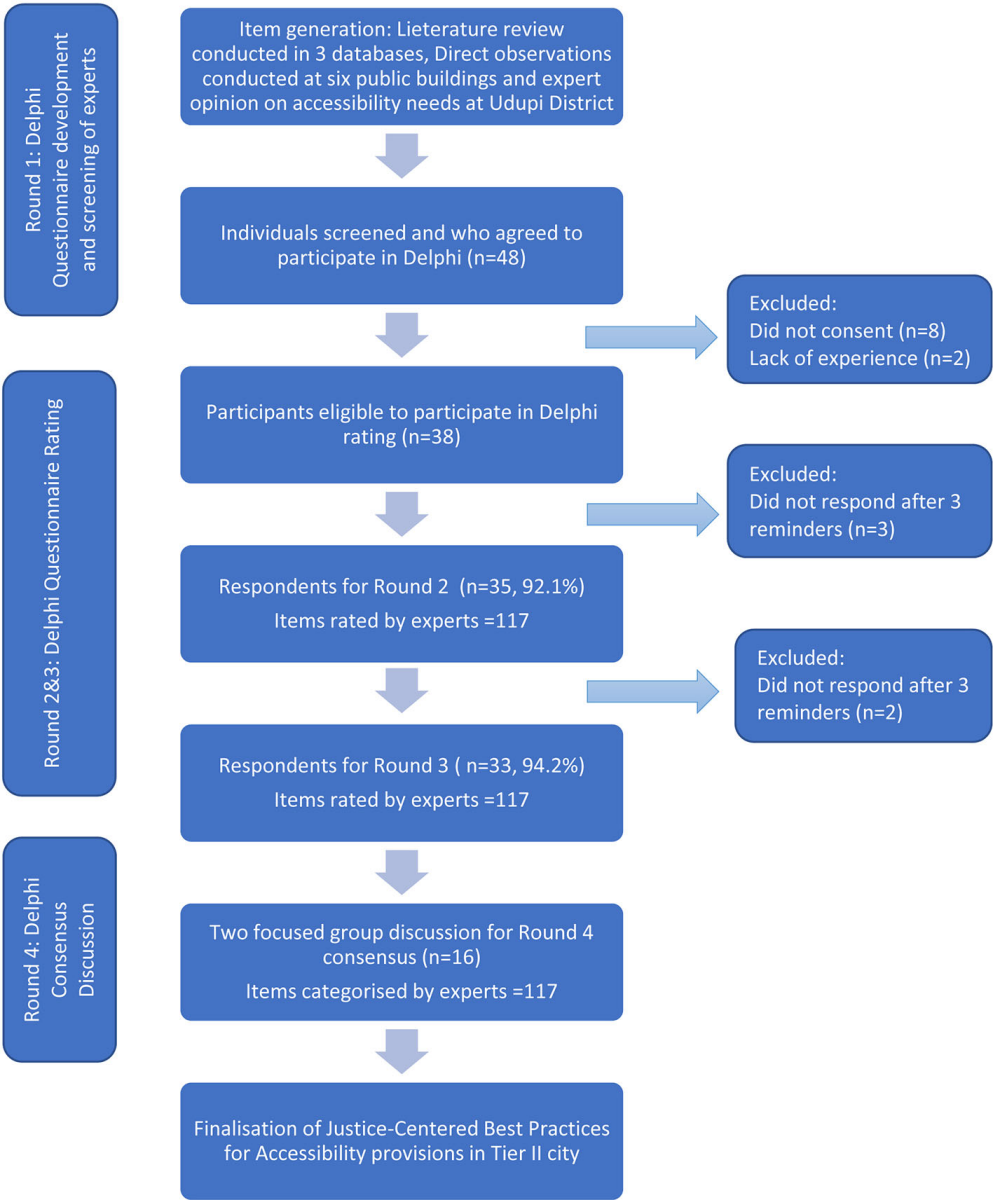
The flow of experts and their response rates in each round of the Delphi process.

Forty-eight individuals, self-identified as experts involved in city development, accessibility, and disability rights, were approached to participate in the study. Following the screening, ten individuals involved in city development were excluded due to low or no experience in the accessibility domain. Experts who did not respond to three reminders at the end of three consecutive weeks were categorized as non-respondents. The flow of experts and their response rates in each round, is displayed in Figure 3.

The individuals involved in the planning and development of the city of Udupi comprised a variety of professionals. Among them were engineers, auditors, and certifiers who were engaged in town planning and urban development. Additionally, policy implementers and executives, including legislative, Commissioners, and public office administrators, who played a significant role in public building developments and inspections of building guidelines, participated in the panel. Furthermore, designers, architects, and structural engineers, who were experts in assessing user needs and planning building designs, were also part of the panel.

The group representing individuals with mobility disabilities was composed of three categories of people. Firstly, individuals with mobility disabilities and disability rights activists were included. Caregivers accompanying individuals with disabilities to public spaces were also part of this panel. Furthermore, professionals involved with persons with mobility disabilities, including community physiotherapists and occupational therapists, also participated. The participants had an average of 7 years of experience in their respective fields, with some having over 20 years of experience in disability-related projects. While the experts primarily practiced in Udupi, it is important to acknowledge that some had experience in other geographical areas as well. Eleven participants were involved in research on accessibility or disability studies including health professionals (n=5), architects (n=5) and engineer (n=1). Eight experts identified themselves with various forms of mobility disability. They were from the disciplines of architecture (n=1), engineering (n=1), rehabilitation workers at the municipality level (n=2), and disability rights activists (n=4). A detailed description of all the experts is given in Table 2.

**Table 2.** Profile and expertise of participants (n=35).

Categories	Frequency (Percentage)	Gender (Male: Female)	Age in years (Range)	Experience in years (Range)	Educational background	Geographical representation	Affiliation	Major role in the Delphi process
Persons with mobility disabilities	7(20)	4:3	25-48	N/A	Varied educational levels (secondary, higher education)	Local (urban and rural areas)	N/A	Provided user feedback based on lived experience
Caregivers of persons with mobility disabilities	5(14.3)	1:4	35-60	N/A	Experience-based knowledge, no formal training in care	Local (urban and rural areas)	N/A	Offered insights on daily challenges and solutions
Disability Rights Advocates	5(14.3)	3:2	35-60	7-12	Degrees in Social Work and Philosophy	Cross-city/state wide	National NGOs, government bodies	Provided policy recommendations, advocacy perspectives
Healthcare Professionals *	5(14.3)	4:1	32-46	5-19	Masters degrees, specialized training in rehabilitation	Regional and national	Private hospital and Higher Education Institute	Provided professional insights and community- oriented perspectives
Architects and Designers	5(14.3)	3:2	35-48	7-23	Degrees in Architecture, certifications in accessibility	Cross-city/state wide	Private firms, architectural consultancies and Higher Education Institute	Provided technical recommendations for design needs and modifications
Contractors, Builders, Engineers, Project Managers	5(14.3)	5:0	35-60	6-28	Engineering degrees, project management certifications	Cross-city/state wide	Private companies, construction firms	Provided feasibility insights, recommendations on structural changes
Commissioners and District Administrators	3(8.6)	2:1	35-42	7-9	Public administration degrees, training in governance	District and state levels	Government bodies, local authorities	Provided administrative perspectives, policy enforcement challenges

Note: N/A: Not Applicable.

* Health Professionals included experts from the disciplines of Physiotherapy, Occupational Therapy, Prothesis, and Orthosis with expertise in neurological, orthopedic, and community rehabilitation.

### Round 1

Item generation

A comprehensive, credible and contextually relevant list of practices and requirements to enhance accessibility to public buildings, a multiple methods triangulation led to generation of an exhaustive 1418 items list. The literature search identified a total of 125 articles relevant to the development of the Delphi questionnaire. After the data extraction process was completed, a total of 862 items were documented under the categories of built environment and non-built environment factors that impact the accessibility of individuals with mobility disabilities.

The onsite unobtrusive observations focused on the contextually relevant factors to be included in the best practices. The common spaces at six public buildings were categorized as external and internal accessible spaces including entrance, interior navigation, facility access, safety measures, services. Observations yielded 231 items for inclusion. Each item was meticulously documented and categorised under built environment and non-built environment factors. Additionally, 12 experts approved to provide a list of requirements for enhancing accessibility. A total of 325 open-ended responses were acquired from the group. Sample codes by experts are given in extended file 5. ^40^

Triangulation and finalisation

The items generated from the three above-mentioned methods were carefully compiled and all duplicates were removed, resulting in a final count of 117 items as given in extended file 6. ^41^ In the subsequent round, the 117 items were subjected to a ranking by experts.

### Round 2

Thirty-eight experts eligible for participation were provided with the questionnaire as shown in Figure 3. Only 35 experts responded (92.1%) on all the items using the scoring sheet with a total score of 15. Forty-five items had a median score ≥13 (Top Priority) and forty-one items had a median score of 12 (High Priority), and only 4 items had median score of 10 (Unsure). No items were considered low scores by the group. Total 69 items had an agreement of more than 80%. Rankings of this round are presented in Tables 3 and 4. The scores are given in the extended file 7. ^42^

**Table 3.** Items with highest ranks and consensus.

Final Rank	Item	Initial Rank in Round 2	Round 2 level of consensus (% High or Top Scores)	Round 3 level of consensus (% High or Top Scores)	Median Round-2	Median Round-3	Final Median Rating in Round 3
1	Staircase Maintenance	1	92.76	93.52	15	15	Top Score
1	Maintenance of Service Areas	2	90.29	91.43	14	14	Top Score
1	Drainage and Surface Maintenance	2	90.29	90.86	14	14	Top Score
1	Community Engagement for Local Authorities	1	90.48	90.86	14	14	Top Score
1	Targeting Attitudes towards Disability	6	89.14	90.48	13	14	Top Score
2	Partnerships with Rehabilitation Professionals	16	87.43	88.00	13	13	Top Score
2	User Involvement in Design Process: Co-creation	27	85.14	85.33	13	13	Top Score
3	Resource Allocation	3	89.52	90.29	13	14	Top Score
4	Lighting Accessibility	3	89.33	90.29	14	14	Top Score
4	Regulatory Compliance	4	88.95	89.33	13	13	Top Score
5	Clear of obstructions	2	89.71	90.29	14	14	Top Score
6	Spatial orientation	5	88.00	88.76	13	13	Top Score
6	Wayfinding Support	5	87.81	88.19	13	13	Top Score
6	Maintenance Protocol-pathways	11	87.05	88.00	13	13	Top Score
6	Electrically Powered Amenities Maintenance	8	87.62	87.24	13	13	Top Score
6	Stakeholder Capacity for Accessibility Projects	9	87.43	86.86	13	13	Top Score
6	Importance of Cost-Effectiveness in Accessibility Solutions	16	85.52	85.90	13	13	Top Score
6	Improving Collaboration for Accessibility Enhancement	11	86.48	85.14	13	13	Top Score
7	Safe to navigate and unrestricted by stray animals	2	89.33	89.33	14	14	Top Score
7	Accessible Signage	7	87.62	88.38	13	13	Top Score
9	Accessible Elevators	1	90.10	90.10	14	14	Top Score
9	Maintenance Protocol-Handrails	9	86.29	86.29	13	13	Top Score
9	Economic Viability of Accessible Solutions	11	85.33	85.33	13	13	Top Score
10	Water and Moisture Resistance	3	87.81	88.00	13	13	Top Score
11	Elevator Maintenance and Usability	2	88.38	88.95	14	14	Top Score
12	Obstacle free entrance	1	88.76	89.14	14	14	Top Score
14	Supportive Handrails	1	87.81	87.24	14	13	Top Score
15	Moveable obstructions	1	87.81	87.81	13	13	Top Score
17	Safety Measures	2	86.86	86.67	13	13	Top Score
20	Ramps for Vertical Access	1	87.05	87.62	13	13	Top Score
20	Maintenance of Ramps	1	86.48	87.43	13	13	Top Score
23	Safe Staircases	2	85.71	85.90	13	13	Top Score
30	Pathway Accessibility	1	86.10	85.33	13	13	Top Score

**Table 4.** Lowest ranked items with scores and consensus levels.

Final Rank	Item	Initial Rank in Round 2	Round 2 level of consensus	Round 3 level of consensus	Median R2	Median R3	Final Median Rating in Round 3	Final Scoring
102	All-Weather Elevator Usability	32	64	84	10	13	12	High Scores
95	Accessible Escalators	30	66	78	10	12	11	High Scores
94	Service Elevators for Accessibility	29	68	84	10	12	11	High Scores
91	Corridor Accessibility and Safety	22	72	86	11	13	12	High Scores
90	Lifts for Vertical Mobility	23	72	79	11	12	12	High Scores
89	Shaded Spaces	17	77	82	11	12	12	High Scores
87	Safety Measures: building entrance opens safely away from road	17	77	71	11	10	11	High Scores
84	Entrance access	11	79	80	12	12	12	High Scores
81	Entrance width	12	79	70	11	11	11	High Scores
79	Surfaces quality	13	78	74	12	11	12	High Scores
77	Immovable obstructions	15	76	78	12	11	12	High Scores
77	Crowd considerations	15	72	70	11	11	11	High Scores
77	Heat Retention Reduction	19	68	78	11	11	11	High Scores
76	Force Requirements	15	72	78	11	11	11	High Scores
75	Thermal Conductivity for Comfort	18	66	83	11	12	12	High Scores
73	Safety, Hygiene and security	14	76	83	11	12	12	High Scores
71	Well-Equipped Staircases	11	79	71	12	11	12	High Scores
66	Drop-off Area Considerations	4	82	82	12	12	12	High Scores
66	Cultural Sensitivity Maintenance	14	70	85	10	13	12	High Scores
63	Platform lifts where space constrains	6	80	83	12	12	12	High Scores
60	Opportunity Cost Reduction in Accessibility Solutions	11	71	85	11	12	12	High Scores
54	Parking Lot Accessibility	3	83	83	13	13	13	Top Scores
54	Handle Accessibility	5	80	74	11	11	11	High Scores
49	Incentivization	9	70	82	11	12	12	High Scores
44	Accessible Service Areas	4	82	84	12	13	13	Top Scores
42	Door Width	3	82	80	12	11	12	High Scores
39	Preventing Falls with Non-Slip Surfaces	2	84	83	13	12	13	Top Scores
37	Public-private partnerships (PPP) for funds and resources	5	78	84	12	12	12	High Scores
34	Safety Measures: broken and uneven pathways	1	85	88	12	13	13	Top Scores
34	Financial Feasibility Assessment-2	2	79	80	12	12	12	High Scores
34	prioritization through policies	1	80	76	12	11	12	High Scores
34	Mitigation of Financial Challenges' Impact	1	78	90	12	14	13	Top Scores

### Round 3

This round was completed by 33 (82%) of experts who participated in Round 2 as shown in Figure 3. Seventy-nine items scored ≥ 80% agreement. None of the item scores when compared between Round-2 and 3 had significant p-value indicating consistency in the scoring. The Kappa value between 0.81 to 1.0 across the 61 items indicates moderate agreement between Rounds 2 and 3, highlighting some consistency in expert ratings over rounds for the group as a whole but also pointing to the need for further discussion. Final ranks with highest and lowest scored items are presented in Tables 3 and 4. Multiple items had tied ranks. The overall ranks secured by 117 items is given in extended file 8. ^43^

Decision tree

To test the consistency and validate the expert consensus from Round 2 and 3, a decision tress analysis was conducted in three stages as shown in Table 5. In the first stage, responses for all 117 items were analysed giving a significant p value of 0.02. This significant difference in the rating between the two rounds indicates a change in the perspectives of the experts during the Delphi process. This implies that the experts’ ratings of the items changed between rounds, potentially due to varying interpretations of the items or evolving perspectives during the Delphi process.

**Table 5.** Decision tree analysis of expert ratings from Round 2 and 3.

Stages	Criteria	Accuracy in % (95% CI)	Kappa value	Sensitivity	Specificity	McNemar’s test p value
1	All 117 items	69.0 (52.9-82.3)	0.29	35.2%	92.0%	0.02 ^*^
2	Kappa value > 80%	62.5 (48.5-75.0)	0.24	57.6%	66.6%	1.0 ^**^
3	Agreement rate > 80%	71.4 (56.7-83.4)	0.42	65.2%	76.9%	0.78 ^**^

Level of significance:

* p value ≤ 0.05 and

** p value ≥ 0.05.

Note: CI= Confidence Interval.

To address this inconsistency, we moved to the second stage of the analysis, which focused on items with a Kappa value greater than 80%. This stage was crucial for isolating items with strong expert consensus and reducing the variability observed in the initial analysis. Here, the p value of 1.0 indicates no significant difference in ratings between rounds for these items, demonstrating that the experts had a more consistent evaluation for items with high Kappa values.

In the final stage, items with an agreement rate higher than 80% were analysed. Focusing on these widely agreed-upon items helped validate and strengthen the expert consensus. Higher accuracy (71.43%) and an insignificant p value of 0.79 suggests improved consistency and agreement among the experts for the items with high agreement rates, thus guiding the consensus meeting towards the final prioritization of items. Results from Decision Tree analysis is given extended file 9. ^44^

### Round 4: Consensus meeting

All 117 items generated with the overall ranks were presented at the final focused group discussion conducted by SM and RS. The top ranked 30 items with agreement of over 80%, were presented separately. Any contradictory or multi-strategy items were rephrased into neutral statements to facilitate discussion. The lowest ranked items were also presented separately. Through the FGDs, the experts worked to refine and group the items, resulting in a final list of items. These were reviewed by 19 panelists (7 advocates and persons with disabilities and 12 experts) in the two FGDs. Some items were coalesced, while others were removed for not reaching the required consensus level. The final consensus statements were crafted to reflect the experts’ agreement on the most critical practices for enhancing accessibility to public buildings for individuals with mobility disabilities Following this meeting, the total number of items reduced from 117 to 86, as 12 items that had failed to reach level of consensus (80%) were now removed and 19 other items were coalesced by the consensus group. All 86 items generated by the Delphi panel were grouped by topic as in the process described above, generating 10 consensus statements summarised in Table 6. Final ranking was generated based on the mean ranking of constituent items.

**Table 6.** Final Consensus statement at the end of Round 4.

Final Rank	Group	Final Consensus statement	Constituent items (n)	Group Mean Rank	Mean Agreement
1	Accessibility Features and Amenities	Provide essential accessibility features and amenities such as lighting, elevators, signage, wayfinding support, electrically powered amenities, ramps, service areas, corridors, and parking lots, incorporating comprehensive spatial and emergency planning.	21	12.90	80
2	Maintenance and Safety	Implement comprehensive maintenance and safety protocols, including regular inspections and updates, to ensure all accessibility features such as staircases, service areas, pathways, ramps, and safety measures are well-maintained and safe for use by individuals with mobility disabilities.	15	12.57	80
3	Technological and Data Solutions for Accessibility	Utilize advanced indigenous technological solutions and robust data collection methods to inform policy decisions, enhance real-time monitoring, and optimize resource allocation for improving accessibility in public buildings.	6	12.46	84
4	User Involvement and Cultural Relevance in Design	Involve users in the design process and ensure that accessibility solutions are culturally and contextually relevant, addressing the specific needs of diverse groups including women with disabilities.	6	12.40	81
5	Stakeholder Engagement and Capacity Building	Foster active engagement and capacity building among all stakeholders, including local authorities, community members, and advocacy groups, to ensure inclusive decision-making and effective implementation of accessibility projects.	11	12.15	82
6	Research and Innovation	Promote continuous research and innovation in emerging technologies, evidence-based practices, and collaborative initiatives to enhance the effectiveness and implementation of accessibility solutions.	6	11.83	81
7	Rehabilitation and Medical Partnerships	Establish strong partnerships with rehabilitation and medical professionals to support and enhance the design and implementation of accessibility solutions.	2	11.81	81
8	Financial Considerations	Secure dedicated funding, conduct thorough financial feasibility assessments, and promote public-private partnerships and incentivization to ensure the financial sustainability of accessibility improvements.	6	11.80	82
9	Policy and Compliance for Accessibility	Ensure strict compliance with accessibility regulations and standards, supported by ethical governance, transparent review methods, and policies that prioritize and mitigate financial challenges to accessibility improvements.	7	11.53	80
10	Environmental and Climatic Considerations	Incorporate environmental and climatic considerations into the design and implementation of accessibility solutions to ensure they are resilient and comfortable for all users.	6	11.32	81

## Discussion

This paper describes a study that sought to generate a list of best practices to guide municipalities, design professionals, advocates, rehabilitation workers, and users of public buildings in identifying approaches to augment accessibility to public buildings. This study represents the first-ever multidisciplinary investigation to scrutinize public building accessibility related practices and methodologies from diverse stakeholders using the Delphi method. Our research has yielded identification of ten priority areas. These areas are discussed under the overarching themes, incorporating the novel insights of people, governance, cost vs. opportunity cost, design process, research and evidence-based implementation, indigenous technological solutions, data on needs, rehabilitation and medical partnerships, user involvement and cultural relevance in design, and capacity building.

### Built and non-built environment considerations and maintenance

The elements of the public building are categorized as internal and external structures. External structures prioritized for immediate accessibility implementation include parking space, access route and entrances. Internal environment structures include doors, sanitary and hygiene and other amenities. Experts particularly highlighted the importance of building entrances and prioritized public toilets among the amenities. Research indicates that these elements are commonly studied in accessibility research. ^13, 21^ Considering the buildings purpose during planning and implementation is an essential step. This will encourage flexible implementation without undue burden.

The importance of maintaining public buildings and spaces in terms of pathways, potholes, climate protection, and stagnation of rainwater were highlighted. These are contextually relevant environmental determinants influencing public buildings accessibility. ^45^ Further, removal of movable barriers like dustbins, stray animals and ensuring accessibility services remain unlocked were also prioritized.

A practical focus is evident in the study participants’ emphasis on immediate, actionable solutions, such as maintaining pathways and ensuring essential areas are free of obstructions. Furthermore, the study’s user-centered perspective adds a novel, practical dimension to broader policy discussions by highlighting the importance of considering the user experience. This perspective underscores the need for real-time solutions, such as ensuring accessibility services remain unlocked and addressing climate protection, which are often overlooked but crucial for daily accessibility.

### Stakeholder engagement

The identified key strategies encompass a shift in the attitudes of both the community and stakeholders, through awareness and advocacy. Unfortunately, disability is still frequently perceived as a state of (in)capability. To create meaningful change at the foundation of this issue, it is crucial to recognize that disability is not a dichotomy, but rather a fluid and evolving process. ^46^ There is increasing acknowledgment that the benefits of accessible built environments are not only limited to advancements of persons with disabilities but extend to the general public. ^47^ It is essential to promote the universal need for accessibility, as doing so will have a positive impact on all levels of society. By increasing visibility and sharing experiences of persons with disabilities, as well as compiling adequate prevalence data, advocates can change the minority perspectives of disability among stakeholders and the general public. ^48^

This paper posits that enhancing comprehension about disability needs could result in companies implementing disability-inclusive practices. Active engagement with the users, with and without disabilities, is required for property managers, ^20, 49^ professionals in the built environment and administrators to implement these practices. ^50^

Furthermore, advocacy and awareness campaigns are instrumental in fostering an empathetic attitude among the people. By cultivating empathy, there is an upsurge in the demand for accessible buildings, which serves as a stimulus for developers and government officials. Users with disabilities can further augment the demand for such buildings by adopting a self-advocacy approach, thereby exerting pressure on the providers. ^51^

Disability inclusive public buildings, have been viewed as an economic burden, aesthetically substandard and catering to minority groups. ^52^ In many jurisdictions that are perceived as an entitlement and not a necessity. According to the Theory of Symbolic Interactionism, actions of people are determined by what they deem important. ^53, 54^ People’s perception of importance is shaped by social and environmental interactions. ^53^ Thus, beliefs of community, administrators, planners and property managers play an important role in accessibility implementation. Encouraging usage of public buildings, enhancement of community involvement and self-advocacies are three strategies recommended to enhance visibility of persons with disabilities. Furthermore, these interactions and strategies will make accessibility a right rather than an entitlement.

### Policies and compliance for accessibility

The implementation of accessibility measures is linked to effective policies, compliance and governance especially at the municipal level. Recognizing disability is a complex phenomenon that is shaped by an individual’s interaction with their physical, structural, social, and political environment is crucial. ^55, 56^ However, a conventional portrayal of disability as a minority and dichotomous representation deviates administrators from realizing that accessibility is a basic human right. Therefore, municipalities must identify users and acknowledge accessibility as a fundamental requirement. Inadequate awareness, lack of data, and a lack of understanding the needs of users with disabilities in public infrastructures, result in ineffective advocacy. Hence, to bring about a systemic transformation and foster a change in perspective, it is crucial for municipalities to engage with all stakeholders. ^57^

Other approaches to promote good governance in the implementation of accessibility measures at the municipal level include encouraging ethical practices, ^58^ collaborating with private organizations and users, promoting coordination among departments, ^57, 59^ and establishing well-defined and measurable plans such as accessibility metrics. ^60, 61^

Collaboration with robust institutions possessing the capacity for advanced research and technology, as well as civic organizations dedicated to promoting a rights-based approach, will lead to the development of more accessible infrastructure and other positive societal transformations. Planners, architects, and engineers operating at the municipal level must prioritize accessibility in both the construction of new public buildings and the retrofitting of existing ones.

Experts emphasized the need for establishment of committees at the municipal level committed to planning and continuous monitoring to break the cycle of conflict that frequently arises between the users of public buildings and municipal administrators. ^59, 62, 63^

The framework considers bridging the gap between preferences of municipal administrations, politicians, planners to that of accessibility needs of persons with disabilities. Economic, aesthetic and structural demands of a public infrastructure may conflict with the accessibility needs. ^57^ Builders and property managers are primarily concerned that accessibility measure may increase the cost of the property. ^17^ Similarly, aesthetic demands of clients may create conflict with the planners’ desire to provide accessibility. Strategies to address these conflicts at private, public administration clients, planners, and implementations level are included in this framework. ^57, 59^

It should be understood that users of public buildings are “faceless”. Thus, planners at municipal level must take diverse groups into account in the planning process. Persons with motor disabilities are only one of many groups among other users in need of accessible infrastructure. According to Saha et al. local government officials encounter challenges with regards to data collection, community engagement, resource allocation, lack of analytical tools, and unclear or complex lines of responsibility. ^63^

### Cost vs opportunity cost

Property developers show reservations to retrofitting and accessibility implementations due to the associated cost. ^64, 65^ Many researchers argue that lack of advocacy within construction industry ^65^ and inability to fully account for the long-term benefits of accessibility retrofitting due to a focus on immediate costs thus contesting this view about the actual cost. ^66^ Even more there are no studies on the opportunity cost incurred due to inaccessible public buildings. Transportation expenditures, healthcare expenditure, lower employment and inadequate access to services are few of the challenges experienced by persons with disabilities.

We propose strategies to reduce economic burden and increase opportunities to participation. Awareness among property managers to understand the long-term value of property over short-term gain through accessibility violation can be provided. Using positive advisements, accessibility as a rating domain under public-building user experience are methods suggested to attract clients and users. Similarly using these techniques, the builders can improve marketability, and commercial profit from the buildings. The suggestion is in line with the challenges reported by planners and property managers in previous studies. ^67, 68^

Successful implementation requires intrinsic and extrinsic motivation. Intrinsic drive includes universalization, rights-based approach, social interactions, and personal/professional experiences. Extrinsic motivation includes positive strategies (e.g. advertisements, user ratings, incentivization) and negative strategies (e.g. audits, penalization) for accessibility implementation. Among all strategies, external audits by national bodies for services and accreditation was deemed a top priority for implementation. ^69^

### Constraints and technology

Space constraints pose significant challenges when retrofitting historical buildings for accessibility. ^70^ As stated by experts, historical buildings often become “thorny issues” when accessibility solutions are introduced due to their architectural and cultural significance. ^71, 72^ Built-area space limitations can impede the incorporation of accessibility features like ramps and elevators, which require substantial external and internal space. For example, constructing a ramp according to building codes may demand a large outdoor area, while installing an elevator might necessitate a considerable underground foundation and specific internal space allocation. ^73, 74^

To overcome these constraints, low-cost and innovative technologies offer potential solutions. Modular ramps, portable lifts, or platform stairlifts can provide accessibility without compromising the building’s structural integrity. Furthermore, historical buildings often face the challenge of balancing modernization with preservation, making value-based conservation critical. ^72^ This approach ensures that the historical and aesthetic value of a building is maintained, while innovative, low-tech solutions are introduced to improve accessibility without altering the original structure excessively.

### Design process

The design of buildings and the design process are a crucial component in the achievement of accessibility. The outcome, which encompasses the design of new buildings or the renovation of existing structures and facilities, must ensure the active participation of users through the process of co-design. ^51^ By using CoDesign, developers can capture and incorporate user perspectives and needs during products, services, or programs development. ^75^ Approaches such as interviews, community walks, user audits, focus groups, and photovoice methods are utilized to document the narratives of users with varying needs. Codesign as a strategy is capable of transforming mindsets, behaviors, empathetic engagement among individuals, planners, and government officials by establishing secure and meaningful avenues for user engagement. ^76, 77^

### Evidence-based collaborative solutions

Inadequate information regarding users with disabilities and the perception of disabilities as a constant phenomenon has led planners and policymakers to assume that there will be low user turnout and high costs associated with accessible solutions for public buildings. ^57^ The lack of data also results in a lack of knowledge, attention, and insufficient implementation of accessibility policies at the municipal level. ^50^ Participatory research with the users throughout the process could alter the approach to accessibility and their requirements in public buildings.

The framework includes research on planning and monitoring accessibility. User audits or surveys with yes/no checklists are commonly used for accessibility evaluation but are costly, time-consuming, laborious, and difficult to interpret. ^78^ Researchers have developed alternative evaluation methods using sensors, drones, robotics, mobile apps, and building information models. ^79, 80^ Municipalities can adopt these efficient and cost-effective tools to improve accessibility data.

The framework also highlights a need for evidence-informed recommendations for public buildings and knowledge dissemination to all the stakeholders. Along these lines, the framework priorities a need for feasible solution. Few strategies under the feasible solutions is to develop indigenous technologies that are affordable, user-centered, and long-lasting indigenous technologies.

### Strength of the study

We adopted a rights-based approach towards accessibility meaningfully involving individuals with disabilities and effective partnerships-with governmental, non-governmental, academia, disabled people’s organization, and civil societies. The methodological strengths lie in its comprehensive, multi-round consensus-building process, diverse stakeholder inclusion, and combining quantitative analysis with qualitative insights, ensuring robust and contextually relevant findings. Triangulation of data sources and adherence to ethical guidelines enhance the study’s credibility. The framework proposed can be adopted as a best practice ensuring specific, measurable and achievable targets for municipalities during planning and monitoring of public projects. The practices proposed can be replicable, in other countries and contexts.

### Limitations

Though the study aim was to develop justice-centered best practices however the proposed practices focus specifically on individuals with motor disabilities. The research does not address the needs of other groups who may also face exclusion, such as older people, people with multiple disabilities, or those affected by gender-based issues. Additionally, while the framework covers many barriers in the built environment, it does not consider cultural or religious practices, such as kneeling or navigating thresholds in religious places, which could also impact access to public buildings.

The use of convenience and snowball sampling techniques may have introduced selection bias, as the sample may not fully represent all stakeholders, limiting the generalizability of the findings. Since the study involved a complex social phenomenon requiring extensive expertise, and a lengthy Delphi process could have impacted the expert’s responses. Also, the geographic focus on Udupi means the results may not be directly applicable to other regions. Despite these challenges, the study provides a well-founded basis for improving accessibility in public buildings for individuals with motor disabilities.

## Conclusion

The priorities identified in this study represent a broad spectrum of crucial areas for municipal authorities, advocacy groups, planners, builders and property managers to address. Proposed strategic interventions range from micro-level actions—such as raising awareness, promoting ethical practices, and fostering motivation—to macro-level measures, including the creation of dedicated committees, the development of comprehensive data collection systems, and ongoing monitoring. This study’s implications extend from individual needs to broader societal impacts, aiming to enhance accessibility and inclusivity in Udupi city. Future research should employ robust methodologies to evaluate the effectiveness of the proposed priorities and their impact on achieving a more accessible and inclusive urban environment.

Despite a trend towards urbanization and increasing population in cities, accessibility still remains a major challenge. Non-inclusive cities impact opportunities for all citizens, disabled and non-disabled. The best practice framework proposed in this study is dynamic and takes a rights-based approach to support local administration to develop policies that tackle exclusion of individuals with disabilities. The strategies highlight that focus on design modifications alone cannot achieve full participation. It should be remembered that for an inclusive city, accessibility is a main pillar to prevent exclusion and attain the Sustainable Development Goals (SDGs).

For international audiences, this study gives a practice framework to initiate, implement and exchange ideas about accessibility at municipal levels. At the national and international level, tier II cities are fast being recognised as emerging places of economic growth. The Government of India’s Smart Cities initiative has highlighted the importance of these cities, which often receive less attention compared to metros and tier I cities. Tier II cities are not as well researched as tier I cities. However, The Smart city initiative aims to equip local authorities with resources to foster inclusivity. Accessibility should be viewed not only through the lens of the built environment, but also in terms of human rights. This includes raising awareness, improving attitudes among planners, understanding opportunity costs, and emphasizing the need for planning, research, collaboration, and advocacy. These aspects are relevant to both national and international agencies.

### Ethical considerations

The study was approved by the Institutional Research Committee, Manipal College of Health Professions, and the Institutional Ethical Committee-Kasturba Hospital, Manipal (IEC 769/2017) to perform the study. The date of approval from ethics committee was received on 14th November 2017. The study period was approved from 14th November 2017 to 30th November 2024. The study was registered under the Clinical Trial Registry- India (CTRI/2018/07/014763). The study adheres to the Declaration of Helsinki. A written informed consent was obtained from experts detailing nature of study and their involvement.

## Data Availability

Figshare: Justice-centered best-practices for accessibility to public buildings in a tier II city: Insights from a Delphi expert consensus: https://doi.org/10.6084/m9.figshare.27084319.v1 ^81^ The project contains the following underlying data:
• Data Set (Total scores in Round 2 and Round 3 by experts). Data are available under the terms of the Creative Commons Attribution 4.0 International license (CC-BY 4.0). Figshare: Extended file 1: Network of experts. https://doi.org/10.6084/m9.figshare.27078994 ^31^ This project contains the following extended data:
• Extended file 1. Network of experts.docx Figshare: Extended file 2: Search strategy, https://doi.org/10.6084/m9.figshare.27079003 ^32^ This project contains the following extended data:
• Extended file 2. Search strategy.docx Figshare: Extended file 3: Scoring sheet, https://doi.org/10.6084/m9.figshare.27079015 ^36^ This project contains the following extended data:
• Extended file 3. Scoring sheet.xlsx Figshare: Extended file 4: Decision Tree Coding, https://doi.org/10.6084/m9.figshare.27079042 ^39^ This project contains the following extended data:
• Extended file 4. Decision Tree Coding.docx Figshare: Extended file 5. Sample codes from experts, https://doi.org/10.6084/m9.figshare.27079045 ^40^ This project contains the following extended data:
• Extended file 5. Sample codes.xlsx Figshare: Extended file 6. Round 1 list, https://doi.org/10.6084/m9.figshare.27079048 ^41^ This project contains the following extended data:
• Extended file 6. Round 1 list.xlsx Figshare: Extended file 7. Round scores, https://doi.org/10.6084/m9.figshare.27079057 ^42^ This project contains the following extended data:
• Extended file 7. Rounds scores.xlsx Figshare: Extended file 8. Overall final ranking, https://doi.org/10.6084/m9.figshare.27079063 ^43^ This project contains the following extended data:
• Extended file 8. overall final.xlsx Figshare: Extended file 9. Result from Decision tree analysis, https://doi.org/10.6084/m9.figshare.27079066 ^44^ This project contains the following extended data:
• Extended file 9. Result Decision tree.docx Data are available under the terms of the Creative Commons Attribution 4.0 International license (CC-BY 4.0). Figshare: CREDES Reporting Guidelines, https://doi.org/10.6084/m9.figshare.27079075 ^28^ Data are available under the terms of the Creative Commons Attribution 4.0 International license (CC-BY 4.0).
