# Supplementary material for: Justice-centered best practices for accessibility to public buildings in a tier II city: Insights from a Delphi expert consensus
Source: F1000Res. 2024 Oct 7;13:1145. [Version 1] doi: 10.12688/f1000research.156920.1 (PMC11803383; doi:10.12688/f1000research.156920.1)
Supplement: Peer review history

# Reviewer report: Reviewer response for version 1

**DOI:** 10.5256/f1000research.172302.r343823

**Published:** 2024-12-30

**Authors:** Adesola C Odole

**Affiliations:** 1 University of Ibadan, Ibadan, Oyo, Nigeria

Thank you for asking me to review this manuscript titled “Justice-centered best practices for accessibility to public buildings in a tier II city: Insights from a Delphi expert consensus” for F1000Research. The study appears original and interesting. It is an attempt to provide a clear guidance for implementing accessible solutions for individuals with mobility disabilities. The methodological rigor for data collection is scientific and sound. However, there are minor comments of interest.

 Please find below specific comments:

 1. Abstract

 -Under Results: The number of identified items for the final list should be specified under feasibility, affordability and priority. The question of concern is what happens if an item scored low on any of the three domains of feasibility, affordability and priority?

1. Introduction (third paragraph)
2. The word between in the sentence “…….. mitigating the symbiotic relationship between poverty, exclusion and disability….” Should be replaced with among.
3. There should be a consistency in the use of either mobility or motor disabilities throughout the entire manuscript.
4. There should be a consistency in the use of either mobility impairments or mobility disabilities throughout the entire manuscript. Authors to check inclusion and exclusion criteria.

2.Inclusion and exclusion criteria

 Was there any specific limit for the experts’ years of experience in advocating for disability rights?

 3. Data collection

 -Why were the instructions given in both English and Kannada? Were the instructions translated from English to Kannada in a standardized manner or interpreted?

 -Did the authors set out to involve specific number of professionals, policy implementers and executives? How many of these individuals were involved in the study?

Is the work clearly and accurately presented and does it cite the current literature?

Yes

If applicable, is the statistical analysis and its interpretation appropriate?

Yes

Are all the source data underlying the results available to ensure full reproducibility?

Yes

Is the study design appropriate and is the work technically sound?

Yes

Are the conclusions drawn adequately supported by the results?

Yes

Are sufficient details of methods and analysis provided to allow replication by others?

Yes

Reviewer Expertise:

Musculoskeletal Physiotherapy, Disability-inclusion, Health outcomes assessment and climate change

I confirm that I have read this submission and believe that I have an appropriate level of expertise to confirm that it is of an acceptable scientific standard.

# Reviewer report: Reviewer response for version 1

**DOI:** 10.5256/f1000research.172302.r343827

**Published:** 2024-12-28

**Authors:** Satendra Singh

**Affiliations:** 1 University of Delhi, New Delhi, Delhi, India

Review of Manuscript

 The authors of this multidisciplinary study sought to generate a list of best practices to guide municipalities, design professionals, advocates, rehabilitation workers, and users of public buildings in identifying approaches to augment accessibility to public buildings. Using a four-round Delphi method, the study identified ten priority areas to establish what they termed Justice-Centered Best Practices (JCBPs) for accessibility provisions for individuals with mobility disabilities.

 The research is highly relevant and timely, especially as the Supreme Court of India is currently deliberating on matters of accessibility, highlighting the inadequate implementation of universal accessibility in India. I approve the manuscript for acceptance subject to the following revisions:

 Major Revisions

1. Introduction:
  - In the second paragraph, include a mention of the United Nations Convention on the Rights of Persons with Disabilities (UNCRPD) as it is a key human rights instrument and has been ratified by the Indian Parliament.
  - Clarify the term "justice-centered perspective" mentioned in the manuscript. Specify whether it refers to distributive justice or disability justice, as both have different implications. Provide relevant references.
2. Figures and Legends:
  - In Figure 2, include the full forms of VRWs and MRWs in the legends or footnotes for clarity.
3. Ethics Committee Approval:
  - The manuscript mentions that machine learning was incorporated later to validate the expert scoring process, which was not part of the initial study protocol. Indicate whether this amendment was communicated to the ethics committee. If not, provide justification.
4. Themes and Coverage:
  - On page 17, the theme of "Built and non-built environment considerations and maintenance" is discussed. However, the paragraph omits details about the non-built environment. Ensure this aspect is addressed. Additionally, while the study focuses on individuals with motor disabilities, sensory aspects (e.g., accessibility of websites) are excluded, which should be clarified.
  - On page 18, in the theme "Policies and compliance for accessibility," emphasize existing legal provisions in the Rights of Persons with Disabilities Act (2016), such as:
    - District-level committees with disabled persons' organizations.
    - Sections 40–45 as the legal mandate for accessibility.
    - India's flagship Accessible India Campaign.
    - Rajive Raturi case in Supreme court seeking stakeholder feedback on accessibility currently
5. Limitations:
  - Acknowledge that the study does not address sensory disabilities (e.g., visual and hearing disabilities) or invisible disabilities (e.g., neurodivergent individuals, those with chronic illnesses, and epilepsy).
  - On page 20, note that the research excludes other marginalized groups, such as older adults, people with multiple disabilities, and those facing gender-based issues.
6. Strategies to Reduce Economic Burden:
  - Under "Proposed strategies to reduce economic burden and increase opportunities for participation," reference provisions mandated in the Rights of Persons with Disabilities Act (2016), such as:
    - No establishment shall be granted permission to build any structure without adhering to government rules.
    - No completion certificate or occupancy permit shall be issued unless accessibility rules are followed.
    - A timeline for making existing public buildings accessible.
    - Accessibility action plan on the website for essential services like primary health centers, civil hospitals, schools, railway stations, and bus stops.
    - Promotion of universally designed consumer products and accessories.
7. References:
  - References 31, 32, and 39–44 are linked to the study supplement. Confirm whether these need to remain in the primary reference list or should be shared separately as supplementary material.

Additional Recommendation

 I strongly agree with the authors' assertion that accessibility is a human rights issue. It should also be framed as a process toward quality improvement. The following reference may be relevant: [ref 1]

 Conclusion

 The manuscript is a valuable contribution to the field, addressing an urgent need to enhance accessibility for individuals with disabilities. Incorporating the revisions suggested above will strengthen its clarity, relevance, and impact.

Is the work clearly and accurately presented and does it cite the current literature?

Yes

If applicable, is the statistical analysis and its interpretation appropriate?

Yes

Are all the source data underlying the results available to ensure full reproducibility?

Yes

Is the study design appropriate and is the work technically sound?

Yes

Are the conclusions drawn adequately supported by the results?

Yes

Are sufficient details of methods and analysis provided to allow replication by others?

Yes

Reviewer Expertise:

Disability justice

I confirm that I have read this submission and believe that I have an appropriate level of expertise to confirm that it is of an acceptable scientific standard.

## References

[1] : Disability inclusion in medical education: Towards a quality improvement approach. *Med Educ* .2023;57(1) : 10.1111/medu.14878 102-107 10.1111/medu.14878 35837829
